# Granzyme family acts as a predict biomarker in cutaneous melanoma and indicates more benefit from anti-PD-1 immunotherapy

**DOI:** 10.7150/ijms.54747

**Published:** 2021-02-06

**Authors:** Xia Wu, Xiaojie Wang, Yan Zhao, Kun Li, Bo Yu, Jianzhong Zhang

**Affiliations:** 1 Department of Dermatology, Peking University People's Hospital, Beijing 100044, China.; 2 Department of Dermatology, Skin Research Institute of Peking University Shenzhen Hospital, Peking University Shenzhen Hospital, Shenzhen, 518036, China.

**Keywords:** granzymes, cutaneous melanoma, overall survival, risk score

## Abstract

The incidence of cutaneous melanoma (CM) increased since the 1970s, and also along with an unfavorable prognosis. CM patients have been verified benefits from immunotherapy, and granzymes (GZMs) comprise more than 90% of the cytolytic granules secreted by cytotoxic T lymphocytes and nature killer cell. Therefore, it is essential to evaluate the prognostic value of GZMs in CM. A total of 633 CM patients was enrolled to access the prognostic value of GZMs. The integrated prognostic value of five GZMs was validated in TCGA-SKCM, GSE65904, GSE53118, GSE19234 and GSE22153 cohorts. GZMscore, age, Breslow's depth and tumor stage are the independent risk factors for CM patients, risk score based on these factors was calculated in TCGA-SKCM and GSE65906 cohorts, which could polarize the CM patients to high- and low-risk groups with diverse prognosis. Patients in low-risk group obtained the activated immune signaling pathways and response, especially for the activated CD8+ T cells, and could benefit more from anti-PD-1 therapy. A higher tumor mutation burden was observed in low-risk group, especially for the mutation of *BRAF*. The protect function of *GZMK* was confirmed by CM cell lines, overexpression of *GZMK* in A375 and G361 cells suppresses cell proliferation, migration, but not cell apoptosis. All in all, we revealed the prognostic value of GZMs in CM patients, which could also act as a predicted value for the selection of responders of anti-PD-1 immunotherapy.

## Introduction

Melanoma is a kind of malignant skin tumor consists of the pigment-producing cells, which could format on other organs, including eyes, ears, gastrointestinal tract, oral and genital mucous membranes 1. Cutaneous melanoma (CM) only accounts for less than 5% of all skin cancers, but caused a greater number of skin cancer-specific deaths 2. There are about 287,723 new cases and 60,712 specific death of CM around the world 3. The non-melanoma cancer of skin accounts for 5.8% of all new tumors, and leads to only 0.7% deaths, while the CM only accounts for 1.6% of all tumor cases, but accounts for about 0.6% deaths 3. In the past decades, the mortality of CM increased sharply, the median mortality of per ten thousand men increased from 1.55 to 2.57, while the mortality of per ten thousand women increased from 1.39 to 1.55 4. The tumorigenesis of CM is affected by both genetic background, including family history and DNA mutations, and environmental factors, including fair-skinned and light-haired persons with high sunburn susceptibility, increased exposure to ultraviolet radiation (UV-A and UV-B rays), and arsenic 5-7. The incidence of CM has increased since the early 1970s in predominantly fair-skinned populations 1. The overall survival (OS) is less to 6 month for the advanced stage of CM patients, and improved since 2011, with the successful treatment of CM with BRAF inhibitor, anti-CTLA-4 therapy, and anti-PD-1 therapy, with a favorable OS result of about 40% 5-year survival rate 8-11. However, only part of the patients could benefit from the above-mentioned therapy, it is necessary to reveal the new biomarkers for the prediction of prognosis and treatment.

Granzymes are a family of homologous serine proteases, which could induce the apoptosis of virus-infected cells or tumor cells accompanied by the perforin 12. Granzymes comprise more than 90% of the cytolytic granules secreted by cytotoxic T lymphocytes and nature killer (NK) cells 13, 14. Immunocytes are the pivotal components for the tumor immune microenvironment, which was also reported as the prognostic marker for cancer 15-17. There are five granzymes (A, B, H, K, M) in humans, each of them contains the specific substrate and executes the different pathways to the function of promoting cell death 18, 19. However, the total value of granzymes family (GZMs) in CM is not clear till to now. In the current study, we evaluated the prognostic value of GZMs in TCGA-SKCM cohort; the prognostic signature was also established and validated in GSE65904 cohort. The potential GZMs impacted signaling pathways, gene mutation, and response to immunotherapy was also extracted.

## Materials and methods

### Patients and datasets

A total of 507 patients diagnosed with CM was enrolled for the current study, 471 of them from The Cancer Genome Atlas (TCGA)-CM cohort, while the other 216 extracted from GSE65904, GSE53118, GSE19234 and GSE22153 cohorts. The mRNA expression profile, gene somatic mutation data, as well as the matched clinical information of TCGA-CM cohort was downloaded from UCSC Xena (https://tcga.xenahubs.net). The clinical information and expression data of GSE65904, GSE53118, GSE19234 and GSE22153 cohorts were obtained from the Gene Expression Omnibus database (https://www.ncbi.nlm.nih.gov/geo/).

### Construction and confirm of the GZMs predicting signature

To combine the effectiveness of five GZMs to CM, we employed the single-sample Gene Set Enrichment Analysis (ssGSEA) analysis, which conducted by the R Bioconductor package Gene Set Variation Analysis (GSVA, v.3.5), to define the ssGSEA GZMs socre (GZMscore) representing the degree of absolute enrichment of the five GZMs in each sample. The GZMscore and other major clinical parameters of CM patients were all enrolled to construct the overall survival (OS) predicting model by multivariate Cox regression analysis. The coefficient of each parameter generated by the multivariate Cox was used to calculate the risk score,


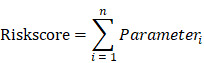


Parameter*_i_* was the value of *i*^th^ parameter. Heatmap was used to show the distribution of each parameter of each patient in the overall cohort. K-M survival analysis was used to indicate the different outcomes of risk groups with the 'survminer' package. The predicting value of the risk score to CM was also confirmed in the GSE65904 cohort.

### Different expression genes (DEGs), gene enrichment annotation and Gene Set Enrichment Analysis (GSEA) analysis

The DEGs between *GZMs* determined high- and low-risk groups were captured by the “limma” package”, the cut-off value was applied with the gene expression fold-change > 1 and *P* value < 0.05. And then, we annotated the DEGs to reveal the activated key signaling pathways Metascape (http://metascape.org) 20. We also conducted GSEA analysis based on the gene expression microarray data, which could detect the significant biological differences between different groups 21. The pathway analyses of training and validation cohorts were performed separately. The enrichment score (ES) is the maximum deviation from zero encountered during that walk, which reflects the degree to which the genes in a gene set are overrepresented at the top or bottom of the entire ranked list of genes. The resulting normalized enrichment scores (NES) is used to adjust the analysis results across different gene sets. The false discovery rate (FDR) q-values were used to adjust for multiple hypothesis testing.

### Tumor immune microenvironment, tumor mutation burden (TMB) and response to immunotherapy in CM patients

The infiltration of 28 immunocytes was assessed by the ssGSEA with the 28 gene sets of immune cell markers, which were previously reported by Charoentong *et al.* 22. The mutation landscape Oncoprint was drawn by R package “maftools” 23. The TMB was also calculated by the “maftools”. To evaluate the individual likelihood of responding to immunotherapy, we employed the Tumor Immune Dysfunction and Exclusion (TIDE) algorithm 24-26. Further, based on a CM cohort that receive anti-CTLA-4 or anti-PD-1 checkpoint inhibition therapy, the specific gene sets were obtained, with 795 genes 27. Subclass mapping analysis was conducted to compare the similarity of the risk groups with the immunotherapy subgroups, aim to point out the responders of anti-CTLA-4 or anti-PD-1 immunotherapy 24.

### Cell lines and cell culture

The melanoma cell lines (A375 and G361) were purchased from the Institute of Cell Research, Chinese Academy of Sciences, Shanghai, China. The A375 and G361 cells were maintained in 1640 medium (Invitrogen, Carlsbad, CA, USA) with 10% fetal bovine serum (FBS) and 1% antibiotics (100 ug/ml streptomycin and 100 U/ml penicillin) at 37 °C in the atmosphere of 5% CO_2_.

### Construction of GZMK overexpression vector and Cell transfection

The GZMK overexpression vector was constructed by Syngentech Co., Ltd. (Beijing, China). Cells were transfected with vectors using Lipofectamine 3000 Transfection Reagent (Invitrogen, Carlsbad, CA, USA) according to the manufacturer.

### RNA extraction and qRT-PCR

TRIZOL (Invitrogen, Grand Island, NY, USA) was used to extract the total RNA from cells, PrimeScript RT Kit with a gDNA Eraser (Takara, Dalian, China) was employed to synthesize the cDNA. Roche light cycler 480 Real-Time PCR machine with the SYBRs Premix Ex Taq (Takara, Dalian, China) was used to perform the Quantitative real-time PCR (qRT-PCR) analysis. The primers were synthesized by Sangon Biotech (Shanghai, China), with the sequence of: *GZMK* forward: ATGCTGGTTAAGCTTCAAACAG, *GZMK*-reverse: GCATTTGGTTCCAGATCTAAGAG; *GAPDH*-forward: CGCTCTCTGCTCCTCCTGTTC, *GAPDH*-reverse: ATCCGTTGACTCCGACCTTCAC. The expression of GZMK in different groups was evaluated by the ∆Ct value.

### Wound healing assay

The migration ability of CM cell lines, A375 and G361 was evaluated by the wound healing assay. Cells were pre-seeded in the 6-well plate to get more than 90% confluence and serum-starved for 24 hours. We scratched the wounds by the sterile 200 μl pipette tips. The wounds were recorded after the infliction of 0 hours, and 24 hours. The quantified results of the wound healing assay were calculated by the covered area to compare to the all wound area.

### Cell proliferation assay

We compared the cell proliferation between *GZMK* overexpression infected group and negative control vector group by cell counting kit (CCK-8) assay (Transgen, Beijing, China). 3×10^3^ cells were seeded in per well in a 96-well plate, at the end time of 0 h, 24 h, 48 h, and 72 h, 10 μl of CCK-8 solution was added to each well and incubated with 2 hours. The reader machine (Bio-Rad, Hercules, CA, USA) was then used to assess the absorbance at 450 nm. Colony formation assay was also employed to evaluate cell proliferation affected by *GZMK* overexpression. 2000 cells/well infected by G*ZMK* vector or NC vector were pre-seeded in 6 cm dishes and incubated for ten days. At the end of the assay, 0.1% crystal violet was added to stain the cells, and then measured the absorbance of 550 nm using a microplate reader.

### Flow cytometry assay

G361 and A375 cells were seeded in 6-well plates. 24H after transfection, the transfected cells were harvested using trypsin without EDTA. According to the manufacturer's protocols, the FITC Annexin V Apoptosis Detection Kit (TransGen, Beijing, China) could double stain cells with FITC-Annexin and PI. The ratio of early apoptotic cells in melanoma cells was determined by the flow cytometry (EPICS, XL-4, Beckman, CA, USA). The experiments were done at least three times.

### Statistical methods and online tools

K-M survival analysis was used to indicate the different clinical outcomes of subgroups with the 'survminer' package, univariate Cox regression analysis was employed to calculate the hazard ration (HR) and 95% confidence interval (95%CI). Comparisons of continuous data between two groups were performed by the Student's T-test or Wilcoxon test. Pearson correlation coefficient test was employed to assess the relationship between two factors. The distributions of categorical variable between high- and low-risk groups were compared by the Chi-square test. For in vitro experiments, all data from at least three independent experiments were presented as mean ± standard deviation (SD). All statistical data were analyzed by SPSS 20.0 software (SPSS Inc. Chicago, IL, USA). A two-sided P value < 0.05 was considered statistically significant. All analyses were performed by R version 3.6.5 (http://www.r-project.org). The prognostic value of *GZMs* in CM patients was also evaluated by GEPIA (http://gepia.cancer-pku.cn/index.html), PROGgeneV2 (http://genomics.jefferson.edu/proggene/index.php) 28 and SurvExpress (http://bioinformatica.mty.itesm.mx:8080/Biomatec/SurvivaX.jsp) 29. The infiltration of immunocytes in wild type or mutated gene subgroup was obtained from the TIMER 2.0 (http://timer.cistrome.org/) 30.

## Results

### GZMs act as the protectors in CM progression

**Figure 1:**
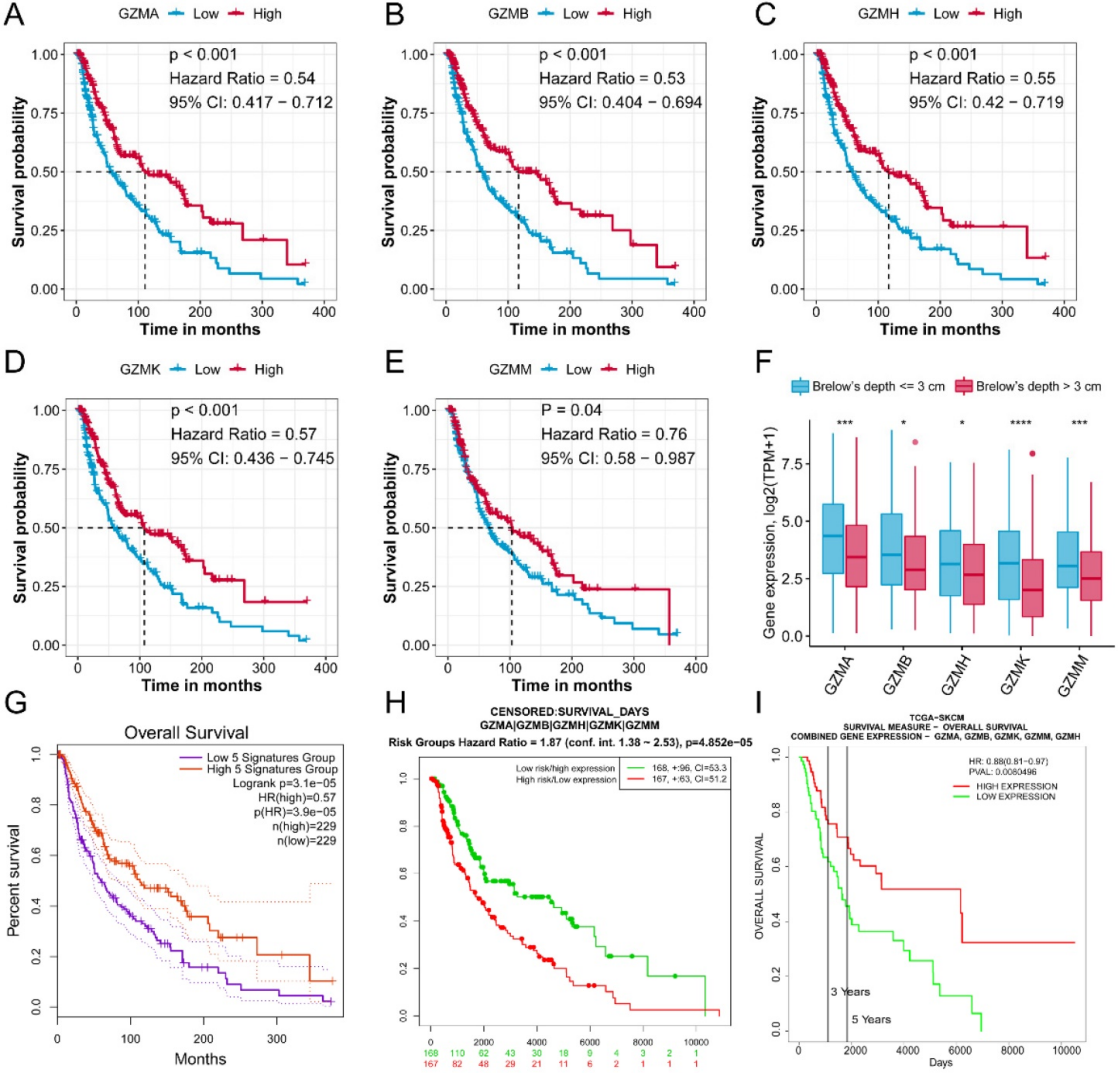
** GZMs are prognostic factors for CM patients**. Low expression of GZMA (A), GZMB (B), GZMH (C), GZMK (D), GZMM (E) indicate poor prognosis for CM patients; (F) Lower expression of GZMs was observed in patients with CM Brelow's depth higher than 3 cm; The combined prognostic value of GZMs validated with GEPIA (G), PROGgeneV2 (H), and SurvExpress (I).

We first evaluated the prognostic value of *GZMs* in TCGA-SKCM cohort. The median expression value of each gene used as the cut-off value to divide patient to high expression and low expression groups. We generated the results that low expression of *GZMA* (HR = 0.54, 95% CI = 0.417-0.712, P < 0.001), *GZMB* (HR = 0.53, 95% CI = 0.404-0.694, P < 0.001), *GZMH* (HR = 0.55, 95% CI = 0.420-0.719, P < 0.001), *GZMK* (HR = 0.57, 95% CI = 0.436-0.745, P < 0.001) and *GZMM* (HR = 0.76, 95% CI = 0.580-0.987, P < 0.001) indicate unfavorable prognosis (**Figure 1A-E**). We also observed the decreased expression of *GZMs* in CM with the Brelow's depth high than 3 cm, which also link the decreased expression of *GZMs* with advanced CM (all, P < 0.05, **Figure 1F**). The combined prognostic value of GZMs was evaluated by online tools, GEPIA (HR = 0.57, P < 0.001, **Figure 1G**), and SurvExpress (HR = 1.87, 95% CI = 1.38-2.53, P < 0.001, **Figure 1H**). We also used the PROGgeneV2 to predict the combined prognostic value of *GZMs* on TCGA-SKCM cohort (HR = 0.88, 95% CI = 0.81-0.97, P = 0.008, **Figure 1I**), GSE53118 cohort (HR = 0.61, 95% CI = 0.43-0.89, P = 0.010, **Figure S2),** GSE19234 cohort (HR = 0.59, 95% CI = 0.36-0.97, P = 0.036, **Figure S3**), GSE22153 cohort (HR = 0.66, 95% CI = 0.49-0.90, P = 0.008, **Figure S4**). These results confirmed the new findings about GZMs to CM prognosis.

### GZMs determined signature acts well to predict the prognosis of CM patients

**Figure 2:**
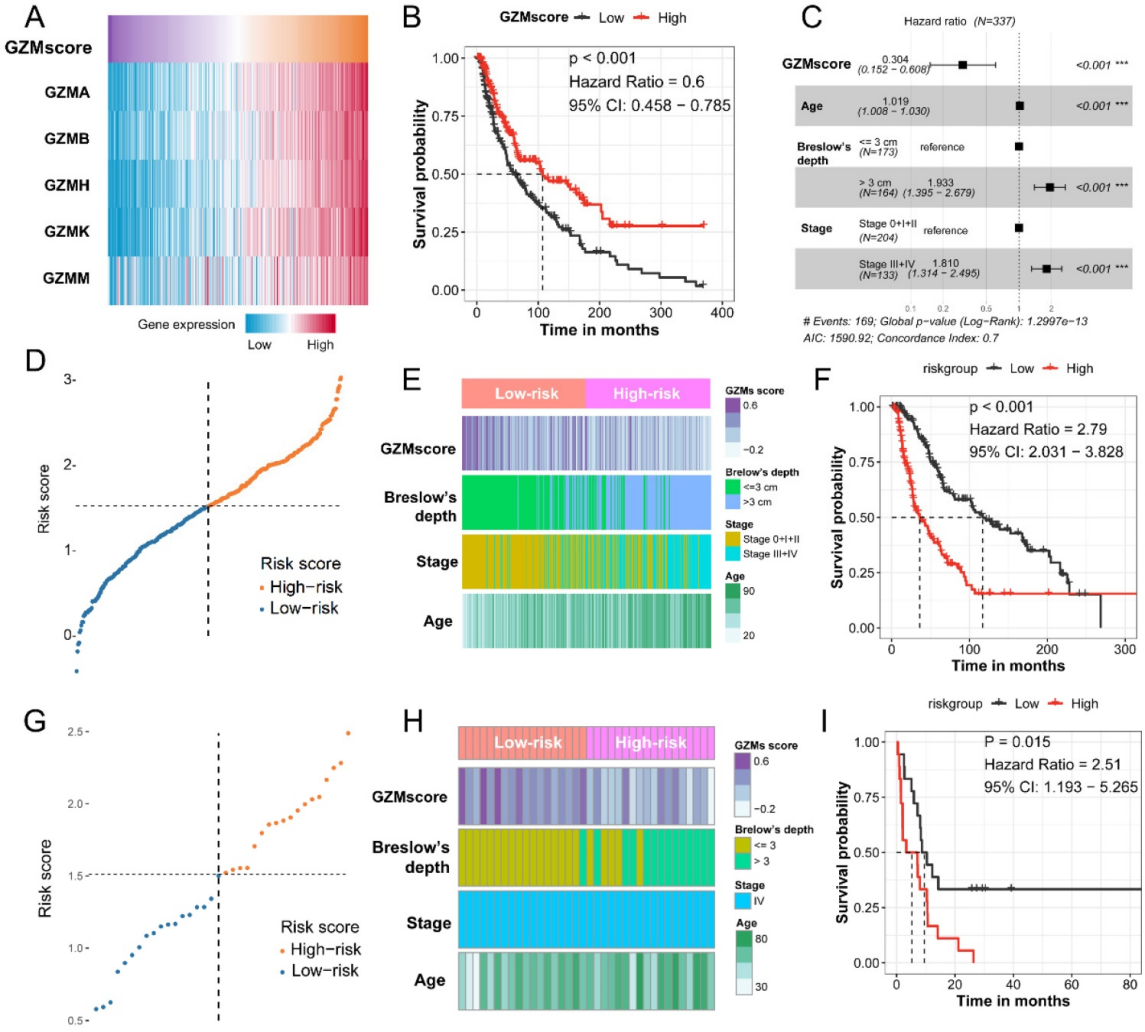
** GZMs and clinical feature determined risk score could predict the prognosis of CM patients.** (A) GZMscore calculated by the expression of five GZMs with ssGSEA; (B) GZMscore could separate the CM patients to favorable and poor prognosis; (C) Multivariate Cox analysis showing the prognostic value of GZMscore, age, Breslow's depth and stage; (D) Risk score calculated by the formula in TCGA-SKCM cohort; (E) Distribution of GZMscore, age, Breslow's depth and stage in high- and low-risk groups of TCGA-SKCM cohort; (F) K-M plot showing the different prognosis in high- and low-risk groups of TCGA-SKCM cohort; (G) Risk score calculated by the formula in GSE65904 cohort; (H) Distribution of GZMscore, age, Breslow's depth and stage in high- and low-risk groups of GSE65904 cohort; (I) K-M plot showing the different prognosis in high- and low-risk groups of GSE65904 cohort.

To combined the value of five *GZMs* expression, we used ssGSEA to generate the GZMscore (**Figure 2A**), lower GZMscore of GZMs predict the poor prognosis of CM patients (HR = 0.6, 95% CI= 0.458-0.785, P < 0.001, **Figure 2B**), which consist with the results in **Figure 1**. With the help of univariate and multivariate Cox regression analysis, we revealed that the GZMscore, Age, Breslow's depth, and tumor stage are the independent risk factors for the prognosis of CM patients (**Table 1, Figure 2C**), the coefficient of each factor was also obtained (**Table 2**). Based on the formula mentioned in the method, we calculated the risk score of each patient in TCGA-SKCM cohort, and separated into high- and low-risk groups (**Table 3**, **Figure 2D**). We observed the high-risk patients contained a lower GZMsocre, more tumors with Breslow's depth, advanced stage (stage III + IV) and higher age (**Figure 2E**), as well as the unfavorable overall survival (HR = 2.79, 95% CI = 2.031-3.828, P < 0.001, **Figure 2F**). To validate the accuracy and stability of the signature, we calculated the risk score of the 36 patients from GSE65904 (**Figure 2G**). The similar tendency reappeared in GSE65904 cohort. Patients in high-risk group shown a lower GZMscore, more with Brelow's depth, and older age (**Figure 2H**). Also, the high-risk group patients linked with poor prognosis of CM patients (HR = 2.51, 95% CI = 1.193-5.265, P = 0.015, **Figure 2I**).

### Overexpressed of *GZMK* could inhibit cell proliferation, migration, but not apoptosis

**Figure 3:**
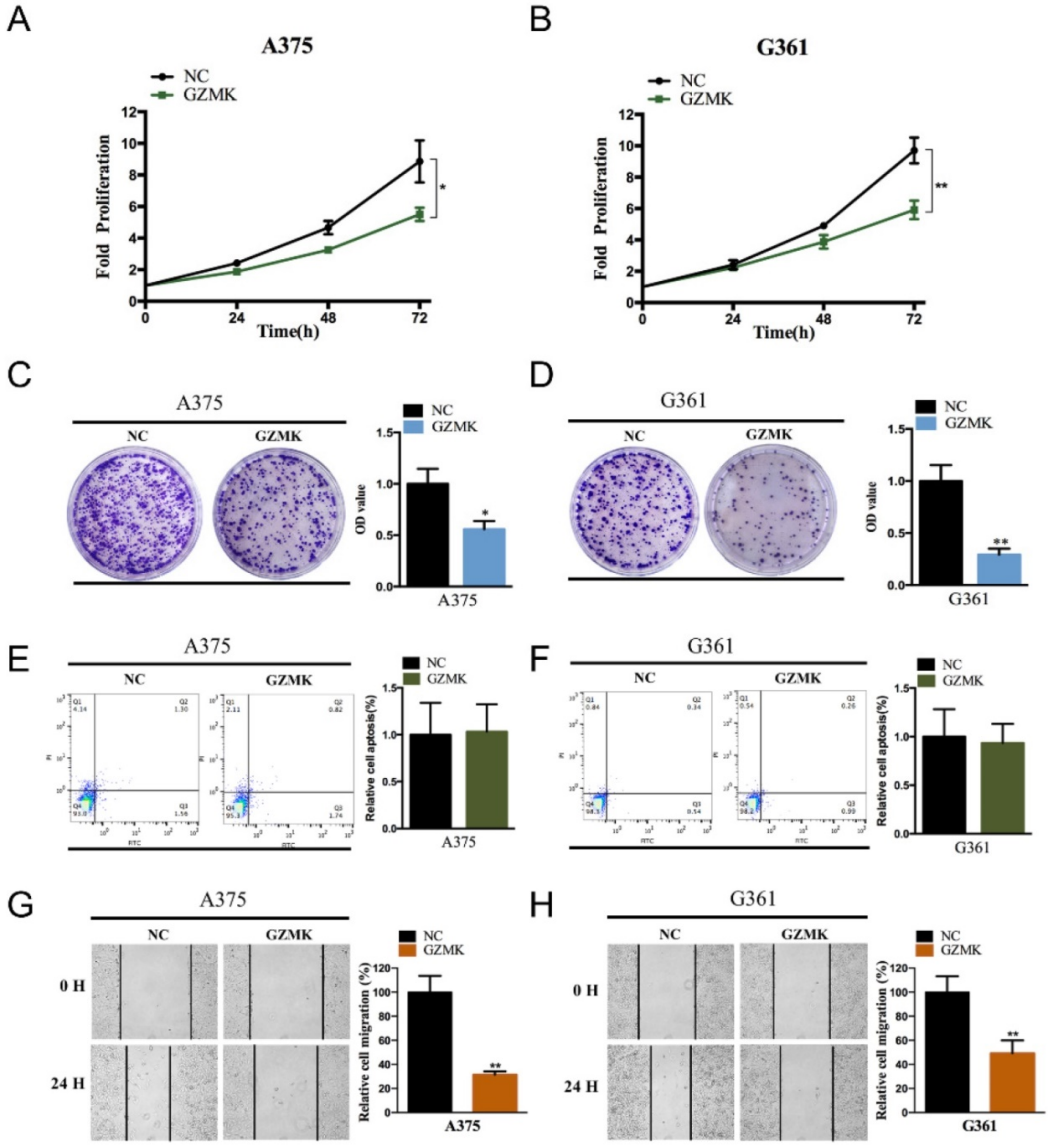
** Overexpression of GZMK suppressed the cell proliferation and migration of CM cells.** Overexpression of GZMK suppressed cell proliferation detected by CCK-8 assay and colony-formation assay in A375 (A, C) and G361 (B, D) cell line; Overexpression of GZMK didn't impact cell apoptosis (E-F); Overexpression of GZMK suppressed cell migration detected by wound healing assay (G-H).

Based on the bioinformatics analysis, we revealed that the *GZMK* acts as a gene suppressor in CM. To validate the function of *GZMK* in cell lines, we first constructed the GZMK overexpressed CM cell lines, the overexpressed *GZMK* was detected by PCR and qRT-PCR in A375 and G361 CM cell lines (**Figure S1A-B**). As compared with the negative control (NC) group, the fold proliferation rate of decreased in GZMK overexpression group in A375 and G361 cell lines (P < 0.05, **Figure 3A-B**). Colony formation assay is another way to evaluate the impact of *GZMK* on cell proliferation, the OD value of GZMK overexpression group significantly lower than that in NC group (P < 0.05, **Figure 3C-D**). As to the effect on cell apoptosis, we didn't revealed the association between *GZMK* and cell apoptosis, the ratio of early apoptotic cells in NC group and *GZMK* group shown a similar distribution in both A375 and G361 cell lines (**Figure 3E-F**). We evaluated the alteration of cell migration after overexpression of *GZMK*, as compared with the 0-hour control, cell migration less in *GZMK* group than NC group (P < 0.05, **Figure 3G-H**). Based on the results generated from in vitro cell experiment, we could obtain the conclusion that the *GZMK* could inhibit CM cell proliferation, migration, but not cell apoptosis, to inhibit the tumorigenesis of CM.

### Different activated signaling pathways among *GZMs* determined high- and low- risk groups

**Figure 4:**
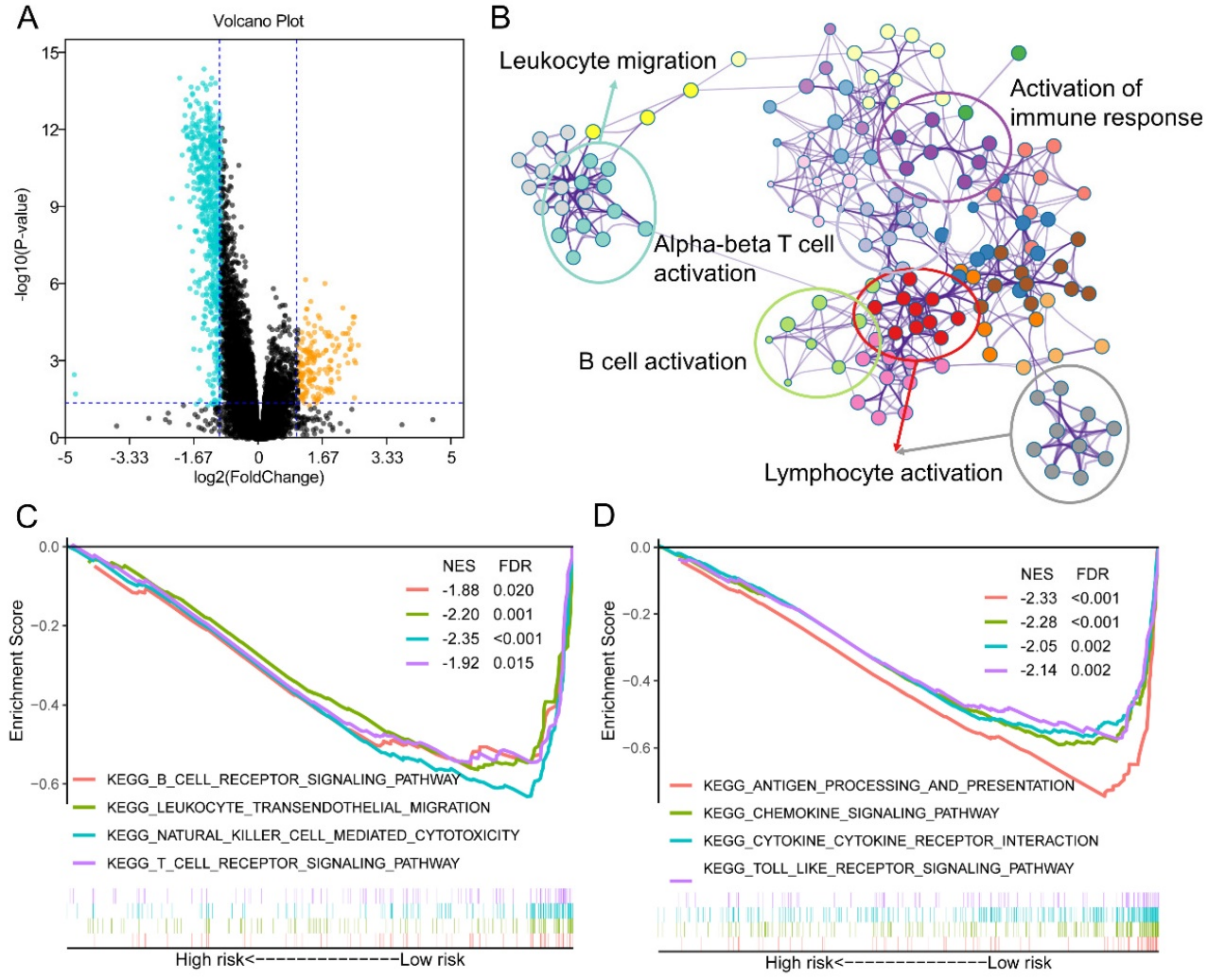
** Different expression genes (DEGs) and signaling pathways between high- and low-risk groups in TCGA-SKCM cohort.** (A) DEGs between high- and low-risk groups; (B) Signaling pathway annotation of decreased DEGs; (C-D) Activated signaling pathways in low-risk group evaluated by GSEA analysis.

To further understand the mechanism of how *GZMs* impact the tumorigenesis of CM, we extracted the DEGs between high- and low-risk groups of CM patients in TCGA-SKCM cohort. With the cut-off value of P value less than 0.05, and log2 (Fold Change) less than -1 or high than 1, a total of 611 decreased DEGs was revealed among the risk groups, as well as another 170 DEGs was observed increasing in high-risk group (**Figure 4A**). The annotation results uncovered that the 611 DEGs enriched in several immune associated signaling pathways, including the biological process of leukocyte migration, lymphocyte activation, B cell activation, alpha-beta T cell activation and the activation of immune response (**Figure 4B**). The GSEA analysis between high- and low-risk group of TCGA-SKCM cohort was also conducted. The low-risk group, which included the high level of *GZMs*, also shown the activated of immunocyte pathways and immune response pathways (**Figure 4C-D**).

**Figure 5:**
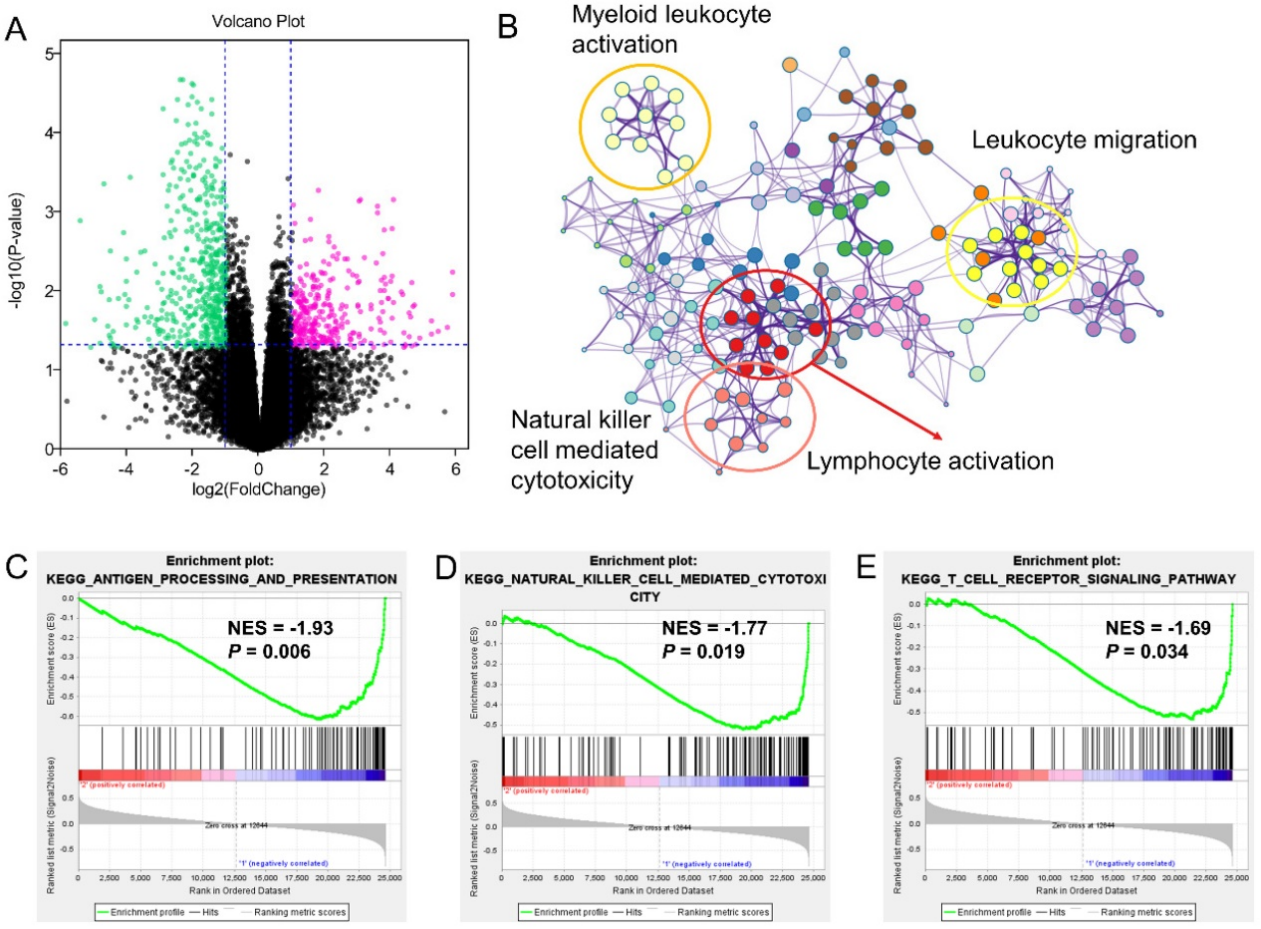
** Different expression genes (DEGs) and signaling pathways between high- and low-risk groups in GSE65904 cohort.** (A) DEGs between high- and low-risk groups; (B) Signaling pathway annotation of decreased DEGs; (C-E) Activated signaling pathways in low-risk group evaluated by GSEA analysis.

The similar findings were generated from the GSE65904 as well. The decreased 549 genes in high-risk groups as compared with low-risk group was enriched in pathways of myeloid leukocyte activation, leukocyte migration, lymphocyte activation and natural killer cell-mediated cytotoxicity (**Figure 5A-B**). The GSEA analysis also pointed out the activation of antigen processing and presentation, natural killer cell-mediated cytotoxicity and T cell receptor signaling pathway in the low-risk *GZMs* group (**Figure 5C-E**). These results supported that *GZMs* positively associated with the activation of immune system and restrict the progression of CM in patients.

### Activated CD8+ cell negatively associated with the increased risk score

**Figure 6:**
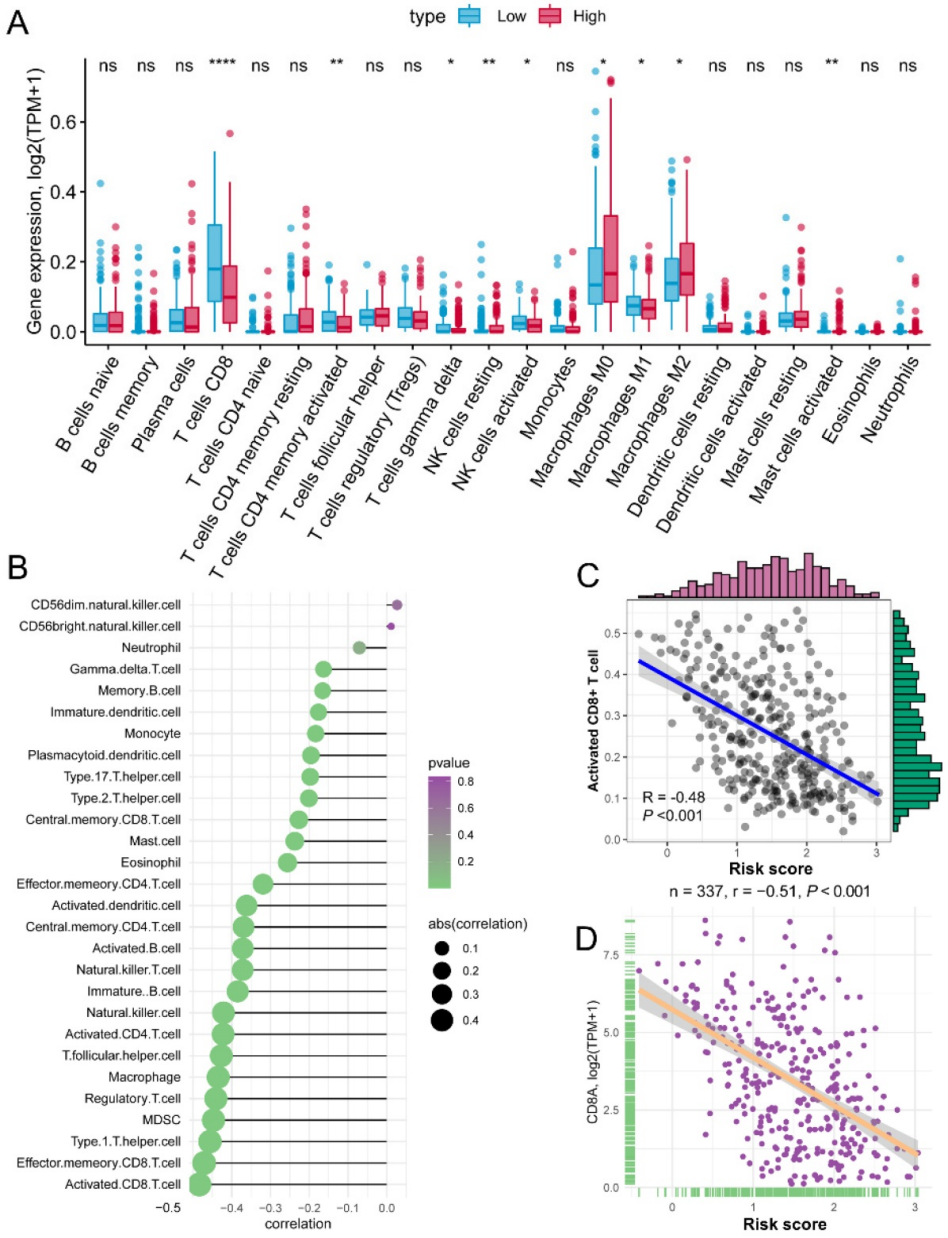
** Risk score is negatively associated with activated CD8+ T cells.** (A) Different infiltration of 22 immunocytes calculated by CIBERSORT between risk groups; (B) Association of risk score with 28 immunocytes calculated by ssGSEA; (C) Risk score negatively associated with the abundance of activated CD8+ T cell; (D) Risk score negatively associated with the expression of CD8A.

The risk score of each patient reflected the combined predict effectiveness of *GZMs* and clinical information. With the results of the annotation of DEGs and GSEA analysis, we drew a line of risk score and immune signaling pathways. Furthermore, we compared the different infiltration of immunocytes between different risk groups. Based on the immunocytes infiltration prediction of CIBERSORT method, patients in the high-risk group shown an increased infiltration of resting NK cells, M0 and M2 macrophages, while the CD8+ T cell, activated memory CD4+ T cell, gamma delta T cell, activated NK cell and M1 macrophages infiltrated more in low-risk group (**Figure 6A**). Meanwhile, we also revealed that the risk score was negatively associated with the infiltration of most immunocytes calculated by ssGSEA of 28 specific types (**Figure 6B**). The activated CD8+ T cell shown the highest association with the risk score (R = -0.48, P < 0.01, **Figure 6C**), as well as the marker of CD8+ T cell, CD8A (R = -0.51, P < 0.01, **Figure 6D**). The positive association of CD8+ T cell with *GZMs* was double confirmed by the TIMER, increased infiltration of CD8+ T cell correlated with the high expression of *GZMA* (R = 0.721, P < 0.01), *GZMB* (R = 0.605, P < 0.01), *GZMH* (R = 0.606, P < 0.01), *GZMK* (R = 0.703, P < 0.01) and *GZMM* (R = 0.289, P < 0.01) (**Figure S5**). These findings suggested that the *GZMs* could inhibit the progress of CM throng the signaling pathways of activated CD8+ T cells.

### The landscape of tumor mutation among GZMs determined risk groups

**Figure 7:**
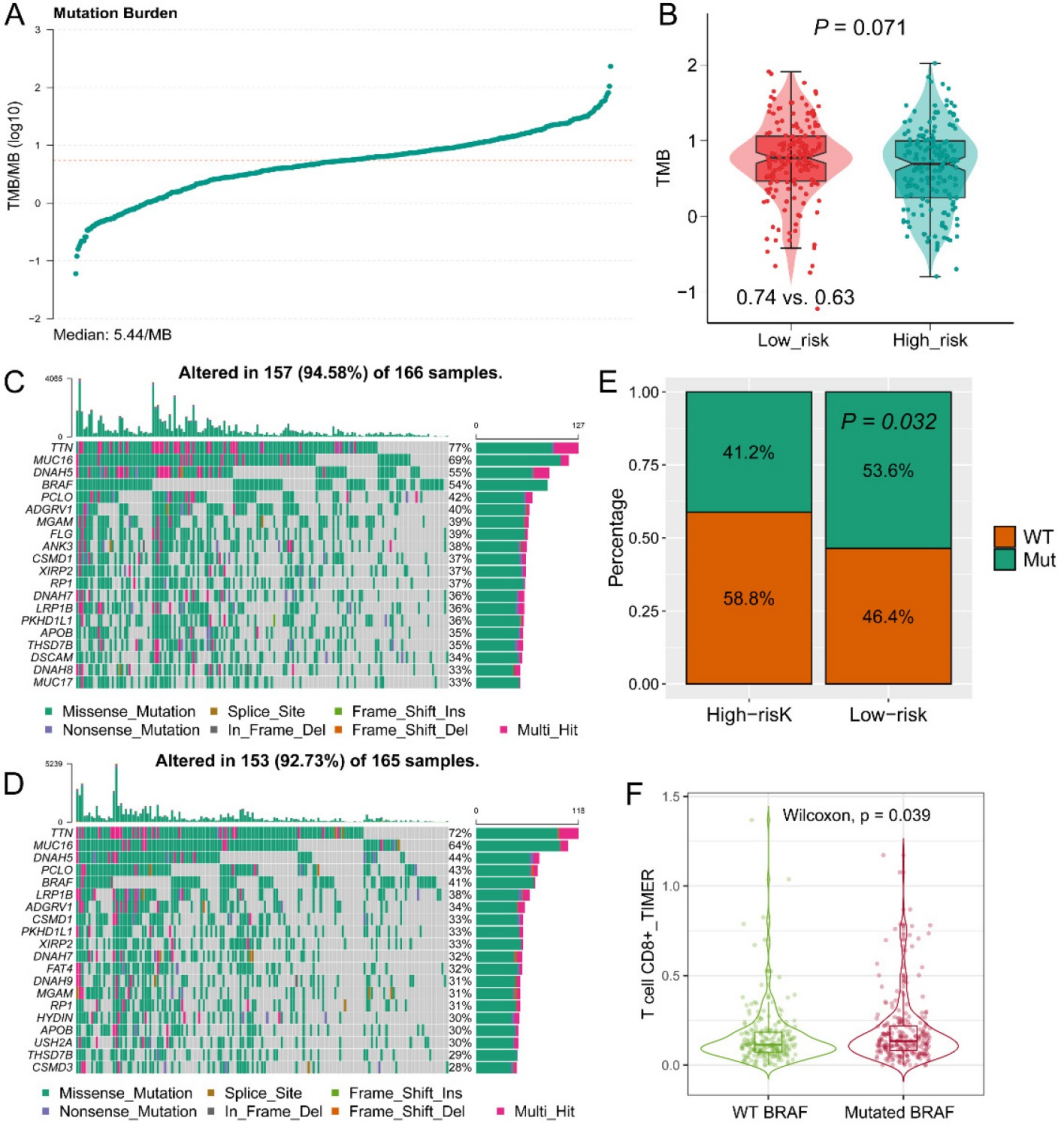
** Tumor mutation burden (TMB) and mutant genes distribution in risk groups**. (A) TMB score of patients in TCGA-SKCM cohort; (B) Different distribution of TMB in high- and low-risk groups; Distribution of mutant genes in low-risk (C) and high-risk (D) groups; (E) More BRAF mutation was observed in low-risk group; (F) Mutated BRAF leads the increased expression of BRAF mRNA.

The TMB value of each patient in TCGA-SKCM cohort was calculated by R package “maftools” (**Figure 7A**). High TMB was observed in the low-risk group (**P = 0.071, Figure 7B**), which is consistent with the findings of immune activation in low-risk group, cause the TMB might stimulate the activation of the tumor immune environment. The top 20 mutant genes in high- or low-risk groups were displayed in **Figure 7C-D**. *BRAF* mutation was observed more in the low-risk group (P = 0.032, **Figure 7E**), and mutated *BRAF* interacted with the increased infiltration of CD8+ T cell (P = 0.039, **Figure 7F**). Therefore, we speculated that the mutated BRAF in low-risk group could stimulate the activation of CD8+ T cells, and lead to a favorable OS.

### The potential responders filtered by the GZMs determined risk score

**Figure 8:**
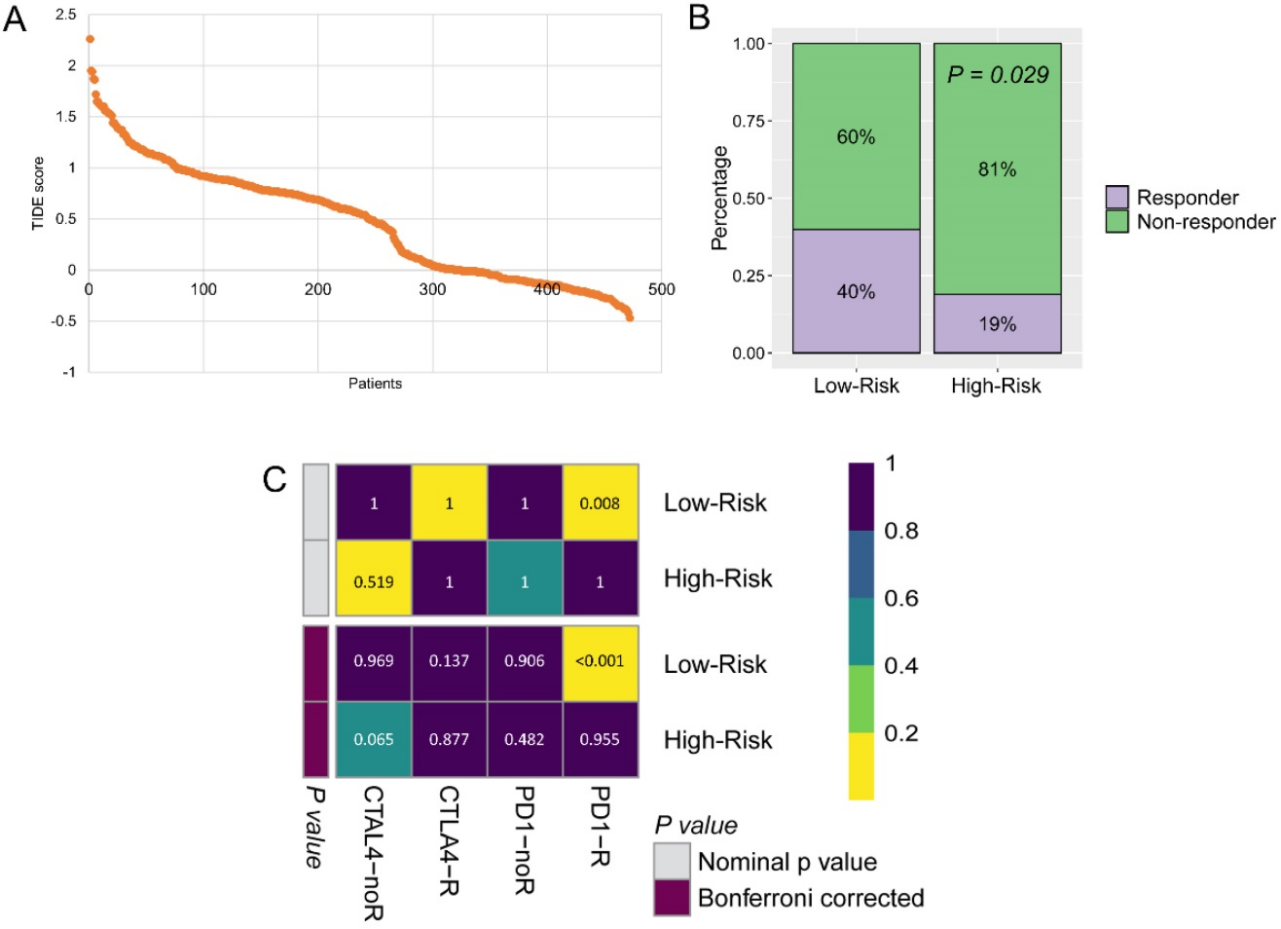
** Low-risk group could benefit more from immunotherapy.** (A) TIDE score of patients in TCGA-SKCM cohort; (B) More immunotherapy responders were observed in low-risk group; (C) Low-risk patients benefit more from anti-PD-1 therapy.

The immunotherapy was widely used for CM patients, because the GZMs risk score is tightly associated with the immune infiltration. We predicted the potential benefit and attempted to find out the responders. The TIDE score for each patient was generated from the online tools (**Figure 8A**), patients in the low-risk group seemed to contain more responders for immunotherapy than high-risk group (40% vs. 19%, P = 0.029, **Figure 8B**). After compared the immunotherapy sensitive genes distribution with the anti-CTLA-4 and anti-PD-1 treated CM cohort, we revealed that patients in the low-risk group could benefit more from the therapy of anti-PD-1, but not anti-CTLA-4 (Bonferroni corrected *P* = 0.008, **Figure 8C**).

## Discussion

GZMs are the encoding genes of the pivotal components, granzymes, of immunocytes. *GZMA* is one of the key genes to quantify the cytolytic activity (CYT) of tumor cells 31, the CYT factor was widely used to measure the activation of immunocytes for the RNA sequence data 32, 33. Shimizu et al. 34 reported that *GZMA* could activate the plasmacytoid dendritic cells and eliciting antigen-specific cytotoxic CD8+ T lymphocytes (CTLs). Rchiad et al. 35 reported the *GZMA* has a tumor suppressor function in T. annulata-infected bovine host leukocytes and in human B-lymphomas. Yang et al. 36 reported that the HCA587 protein vaccine could eradicate the CM by increasing the expression of *GZMB* by CD4+ T cells, which is also a cooperate function of IFN-γ. Wu et al. 37 reposted that *PFN, GzmA, GzmB, GNLY* are the co-factors that affect the NK-extracellular vesicles mediated cytotoxicity to tumor cells, *GzmA* could inhibit the tumor cells through a caspase-independent death pathway. The prognostic value of GZMs family has not been illustrated till now.

In the current study, we evaluated the prognostic value of the five *GZMs* in CM patients. Decreased GZMs indicated the unfavorable OS, we revealed and confirmed the findings based on TCGA-SKCM cohort, GSE65904 cohort, GSE53118 cohort, GSE19234 cohort and GSE22153 cohort. Patients with the low-risk score determined by GZMs shown an activated status of immune-associated signaling, such as immune cell activation, immune cell migration and antigen presentation. Activated CD8+ T cells are the most impacted by *GZMs*. These results suggested us that the altered expression of *GZMs* could impact the progress of CM through CD8+ T cell-mediated immune response, CM patients with a poor prognosis might be caused by the exhausted of immune infiltration. In a further study, we predict the potential responders in high- and low-risk group. More immunotherapy responders were extracted in the low-risk groups, which mean with the high infiltration proportion, and in the contrary, high-risk group patients benefit less from the immunotherapy. Lymphocyte infiltration and abundant cytokines are the key features for the effective treatment of immune checkpoint inhibitors (ICI) therapy, the lack of T cell in the tumor microenvironment indicated the characteristics of a “cold tumor” 38. In the subsequent predict of response to anti-CTAL4 and anti-PD-1 therapy, there is no doubt that the low-risk group patients could benefit more from the anti-PD-1 therapy.

What's more, we compared the distribution of mutated genes among two risk groups and revealed that *BRAF* mutation was observed more in the low-risk group, and mutated *BRAF* interacted with the increased infiltration of CD8+ T cell. The mutation of *BRAF* in CM patients was widely studied. Davies et al. 39 reported that the somatic *BRAF* mutation was detected in about 66% malignant tumors, including CM. Beuer et al. 40 concluded that age, anatomic site and degree of sunburn are the independent factors affect the frequency of *BRAF* mutation in CM. Vemurafenib is a specific therapy for the *BRAF* mutant CM patients, it could inhibit the ERK phosphorylation and causes the cell death of CM, which was approved by the US Food and Drug Administration 41. In the current study, we revealed that patients in the low-risk group have a high rate of *BRAF* mutation, therefore, these patients are the ideal ones to receive the vemurafenib treatment.

In summary, five GZMs are suppressors in CM tumorigenesis, GZMs could activate the immune response and prolong the OS of CM patients, patients' with the lower GZMscore could benefit more from anti-PD-1 therapy and vemurafenib treatment. Overexpression of GZMK could suppress the proliferation and migration, but not cell apoptosis in CM cell lines.

## Supplementary Material

Supplementary figures.

## Figures and Tables

**Table 1:** The predicted value of GZMscore and clinical features to CM patients

Parameters	Univariate Cox Analysis			Multivariate Cox Analysis		
	HR	95%CI	*P* value	HR	95%CI	*P* value
GZMscore	0.6	0.46-0.79	2.00×10^-4*^	0.3	0.15-0.61	7.62×10^-4*^
Age	1.02	1.01-1.03	2.28×10^-5*^	1.02	1.01-1.03	9.11×10^-4*^
Gender (Male vs. Female)	0.944	0.69-1.30	0.721			
Brelow's depth (> 3 cm vs. ≤ 3 cm)	2.49	1.82-3.42	1.53×10^-8*^	1.95	1.40-2.71	7.42×10^-5*^
Stage (III+IV vs. 0+I+II)	2.01	1.47-2.76	1.39×10^-5*^	1.81	1.31-2.49	2.86×10^-4*^

*, P <0.05.

**Table 2:** The coefficient of GZMscore and clinical features to the signature

Parameters	coef	exp (coef)	se (coef)	z	P value
GZMs score	-1.19228	0.30353	0.354172	-3.366	7.62×10^-4*^
Age	0.01838	1.01855	0.005542	3.317	9.11×10^-4*^
Brelow's depth (> 3 cm vs. ≤ 3 cm)	0.659245	1.933332	0.166378	3.962	7.42×10^-5*^
Stage (III+IV vs. 0+I+II)	0.5936	1.810494	0.16362	3.628	2.86×10^-4*^

Co-ef, co-efficient; Exp (co-ef), Expectation (co-ef); Se (co-ef), standard error (co-ef); *, P < 0.05.

**Table 3:** The Distribution of clinical features among low- and high-risk group

	TCGA-SKCM			GSE65904		
	Low-Risk	High-Risk		Low-Risk	High-Risk	
Age, years old	53.51±15.32	64.44±13.65	<0.001*	61.00±14.71	66.28±13.84	0.275
**Gender**			0.092			0.74
Male	112	97		8	10	
Female	56	72		10	8	
Breslow's depth			<0.001*			<0.001*
**≤3**	140	33		17	5	
**>3**	28	136		1	13	
**Stage**			<0.001*			-
0+I+II	139	104		-	-	
III+IV	29	65		18	18	
GZMscore	0.21±0.24	0.03±0.20	<0.001*	0.41±0.16	0.14±0.22	<0.001*

*, P < 0.05.
