# Supplementary material for: Granzyme family acts as a predict biomarker in cutaneous melanoma and indicates more benefit from anti-PD-1 immunotherapy
Source: Int J Med Sci. 2021 Feb 6;18(7):1657–69. doi: 10.7150/ijms.54747 (PMC7976569; doi:10.7150/ijms.54747)
Supplement: Supplementary file 1 — Supplementary figures. [file ijmsv18p1657s1.pdf]

Figure S1. Detection of GZMK expression in A375 and G361 CM cell lines by PCR and qRT-PCR.

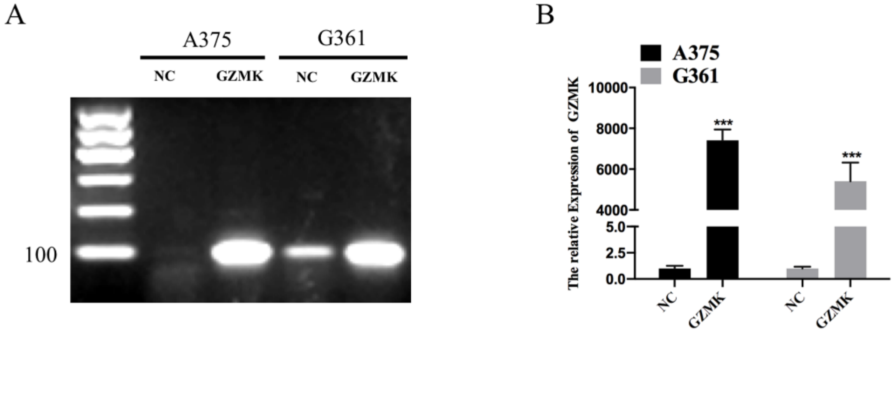

Figure S1

Figure S2. Integrated prognostic value of GZMs in GSE53118 cohort.

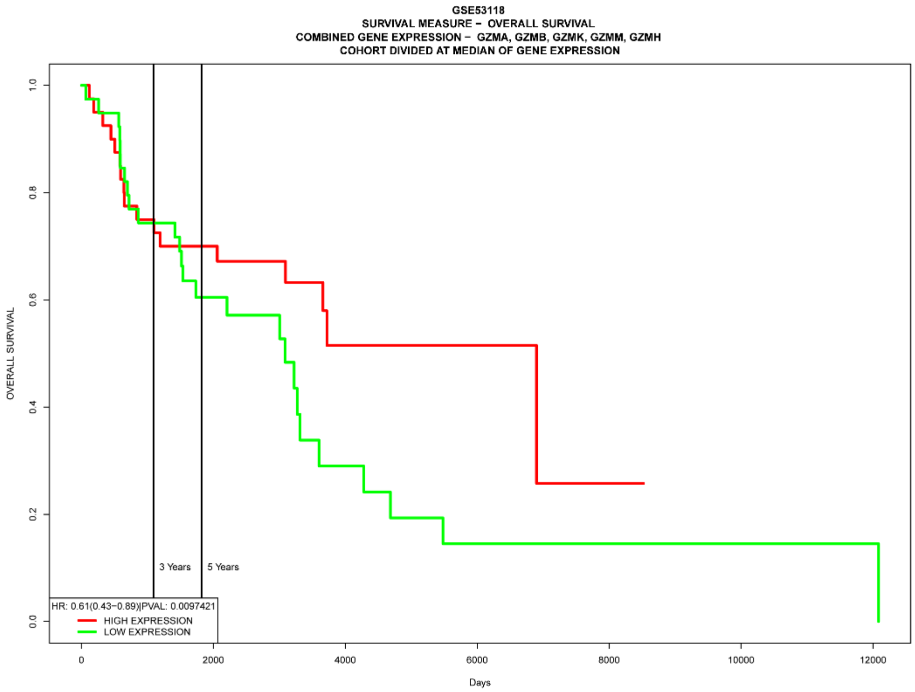

Figure S2

Figure S3. Integrated prognostic value of GZMs in GSE19234 cohort.

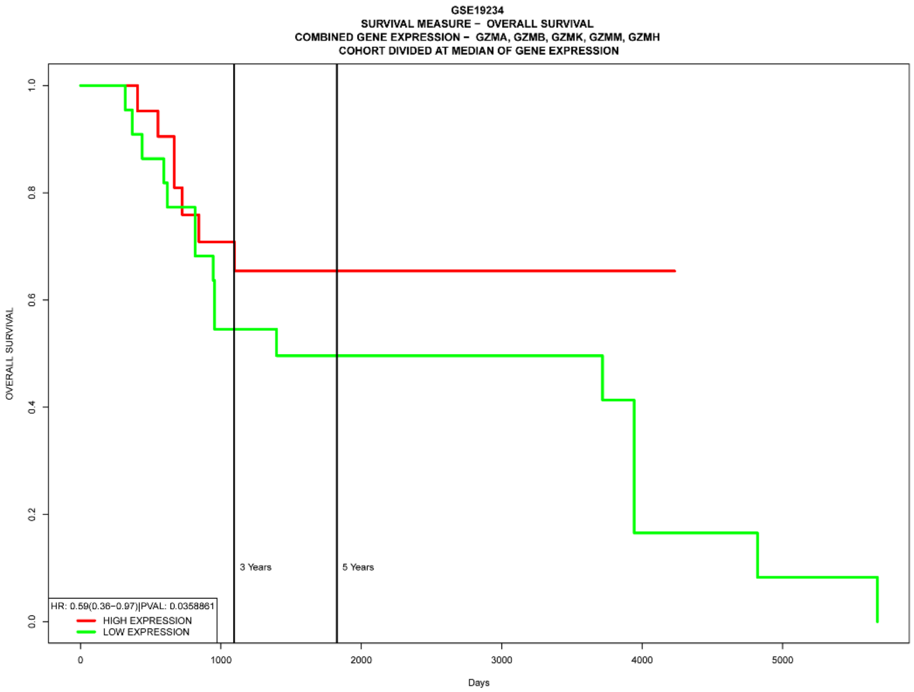

Figure S3

Figure S4. Integrated prognostic value of GZMs in GSE22153 cohort.

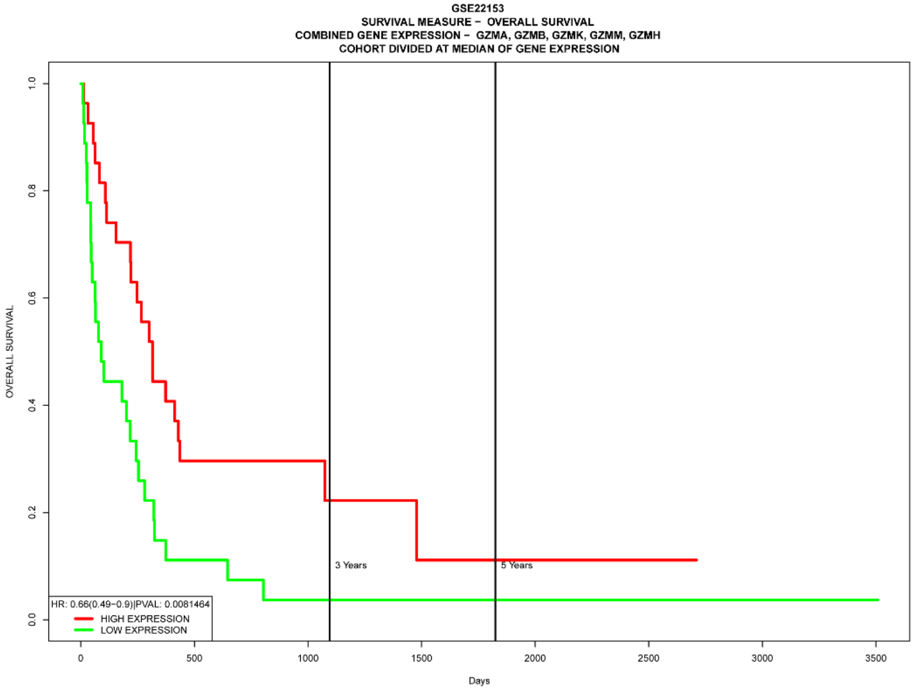

Figure S4

Figure S5. GZMs expression are positively associated with the infiltration of CD8+ T cells.

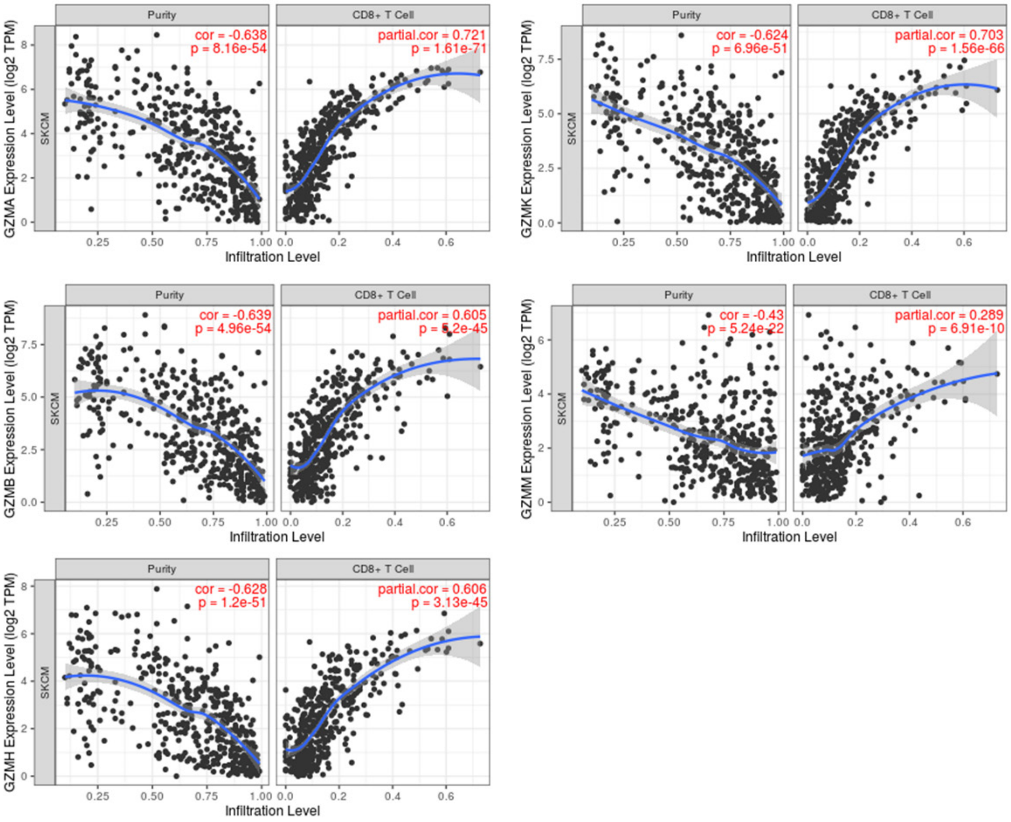

Figure S5
